# Trophoblast retrieval from the cervical canal to predict abnormal pregnancy early in gestation: a pilot study

**DOI:** 10.1186/s12884-023-05499-4

**Published:** 2023-03-18

**Authors:** Xiaoke Yang, Liuyezi Du, Yue Li, Lin Liang, Linlin Ma, Shaowei Wang

**Affiliations:** 1 Department of Gynecology and Obstetrics, Beijing Hospital, National Center of Gerontology, Institute of Geriatric Medicine, Chinese Academy of Medical Sciences, Beijing, P.R. China; 2 Graduate School of Peking Union Medical College, Beijing, P.R. China; 3 Beijing USCI Medical Laboratory, Beijing, P.R. China

**Keywords:** Non-invasive prenatal testing, NIPT, cfDNA, Trophoblast, Cervix

## Abstract

**Background:**

The current detection of fetal chromosomal abnormalities by non-invasive prenatal testing (NIPT) mainly relies on the cell free DNA(cfDNA) in the maternal blood. However, a gestational age of less than 12 weeks or a high maternal BMI affects cfDNA fetal fraction and further the detection by NIPT negatively. In this study, we aim to retrieve the trophoblast cells from the maternal cervix to develop a new sampling method for NIPT enabling an earlier use of NIPT.

**Methods:**

We enrolled three patients who wanted to undergo induced abortion at Beijing Hospital between January 2022 and March 2022. Peripheral blood, cervix specimen, and the abortion tissue were collected and processed for each patient. Allele frequencies of the mutated gene loci of the maternal blood and the cervix sample were compared and the Sex Determining Region Y (SRY) gene was tested.

**Results:**

The allele frequencies of the mutated gene loci showed no significant difference between the maternal blood and the cervix sample. But we successfully detected signal of the SRY gene in the cervix sample of the only patient carrying a male fetus.

**Conclusions:**

The detection of the SRY gene in a cervix sample indicated a successful retrieval of trophoblast cells from the cervix canal. Further study needs to be conducted to verify our finding before its application to the clinical settings.

## Background

Chromosomal abnormalities such as trisomies and sex chromosomal abnormalities may negatively affect fetus and cause arrested embryo growth, organ abnormalities, or even death in utero [1, 2]. Traditional non-invasive methods for detecting abnormal chromosomes such as ultrasound and prenatal serum screening may cause a high proportion of false negatives and false positives. Whereas, invasive testing methods for accurate diagnosis such as chorionic villus sampling are expensive and may lead to miscarriage [3].

The discovery of fetal DNA in the cell-free plasma of pregnant women by Lo et al [4] and the development of next-generation sequencing (NGS) based methods enabled more accurate screening of fetal chromosomal abnormalities by non-invasive prenatal testing (NIPT). However, studies have found that an early gestation or a high maternal BMI may lead to an unsatisfactory cell free DNA(cfDNA) fetal fraction. Thus, NIPT is normally conducted after a gestation of 12 weeks and its performance for pregnant women with a high BMI is not very satisfactory [5].

Since the discovery of cfDNA, several collection methods from different parts of the genital tract have emerged, they include cotton swab insertion, endocervical mucus aspiration, and intrauterine or endocervical lavage [4, 6–8]. These techniques require a deep insertion of sampling material into the genital tract of pregnant women, which present some degree of invasiveness. In 2014, the Armant group presented the “Trophoblastic Retrieval and isolation of Cervix” (TRIC) method. This method collects endocervical samples between 5 and 20 weeks of gestation by inserting a brush approximately 2 cm into the endocervical canal, followed by rotations to trap mucus [9].

Other studies also have shown that trophoblast cells could be collected through the cervix as early as 5 ~ 7 weeks of pregnancy [10]. In our pilot study, we aim to retrieve the trophoblast cells from the cervix canal to explore a new sampling method for NIPT. The successful isolation of trophoblast cells from the cervix canal early during gestation will enable an earlier application of NIPT to detect chromosomal abnormalities.

## Methods

### Ethical approval

The study was approved by the institutional review board of Beijing Hospital. Written informed consent from all the patients were obtained. Qualified professionals ensured that all the contents of the informed consent form were read and understood before signing.

### Accordance statement 

All methods were performed in accordance with the relevant guidelines and regulations.

### Study participants

Between January 2022 and March 2022, we enrolled three patients who wanted to undergo induced abortion at Beijing Hospital. The women were aged 31, 35, 44 years old with a gestation of 11.9, 7.3 and 8.3 weeks, respectively. The BMI was 17.6, 21.6 and 23.4, respectively.

### Inclusion criteria

- (1) Women between 5 and 12 weeks of gestation
- (2) Intrauterine pregnancy confirmed by ultrasound
- (3) Women who planned a termination of pregnancy
- (4) No trichomonas or mycobacterial infections and vaginal cleanliness degree I (determined by routine vaginal discharge tests)
- (5) Absence of acute or chronic inflammation of the cervix
- (6) No fever or vaginal bleeding during pregnancy
- (7) No sexual intercourse in the last five days
- (8) No history of using drugs to promote uterine contraction or prostaglandin-like drugs to promote cervical maturation during pregnancy
- (9) Voluntarily participated in this study

### Exclusion criteria

Women who have any contraindications to abortion in early pregnancy: (1) acute phase of any disease requiring hospitalization after treatment; (2) genital inflammation; (3) systemic condition unable to tolerate surgery; (4) body temperature ≥ 37.5℃ twice 4 h apart before surgery.

Note: The gestational age of the subjects was extrapolated using the last menstrual method and ultrasound method. If the embryo length is sucessfully measured by ultrasound, gestational week = embryo length (cm) + 6.5, if not, gestational week = maximum internal diameter of the gestational sac (cm) + 3. To define the early pregnancy embryonic arrest specimen, the ultrasound needs to indicate that the embryo had stopped developing (no heart tube pulsation, no germ visible, etc.).

### Sample collection

For each woman, after informed consent, 10 ml of peripheral blood, a specimen collected from the cervix, and the abortion tissue were obtained. Cells from the cervical canal were obtained noninvasively with a special cytobrush before performing manual negative pressure aspiration. Chorionic villus tissue was obtained aseptically during the abortion for routine chorionic villus cell culture. The collected exfoliated cell specimen was washed in sterile saline. A large amount of cervical mucus was mixed in the specimen, and the cervical mucus in the specimen was obtained by adding appropriate amount of collagenase digestion.

The cervix samples were all collected before termination of pregnancy(TOP). The blood of patient A was collected soon after TOP and the blood of patient B and C were collected before TOP.

### Specimen processing

The genomic DNA (gDNA) was extracted from the cervix samples using QIAamp DNA Blood Kit (Qiagen, Hilden, Germany). For the blood and abortion samples, we isolated the gDNA with MagPure Tissue DNA LQ Kit (Magen, Guangzhou, Guangdong, China). The gDNA was fragmented to around 200 bp by Qsonica Sonicator, after which the gDNA library was constructed by VAHTS Universal Pro DNA Library Prep Kit for MGI (Vazyme, Nanjing, Jiangsu, China). xGen CNV Backbone Hyb Panel (IDT, Coralville, Iowa, USA) was used to capture the target regions of gDNA libraries. The post-capture libraries were sequenced on MGISEQ-2000 platform (MGI, Shenzhen, Guangdong, China) with 100 bp paired-end sequencing strategy. All procedures were performed according to relevant guidelines and manufacturers’ instructions.

### Statistical analysis

The statistical analysis was conducted for each individual separately.

#### Selection of gene loci for analysis

The main challenge of this method is to diffrentiate the signal of the fetus from the mother’s in the cevix. If a mutation happens in the mother and her fetus at the same time, conclusion cannot be drawn that the mutation detected in the cervix represent the mutation in the fetus. To solve this problem, we filtered each gene loci with a different mutation type in the mother and her fetus, which is a exclusive signal for the fetus, coming from the paternal origin.

**Fig. 1:**
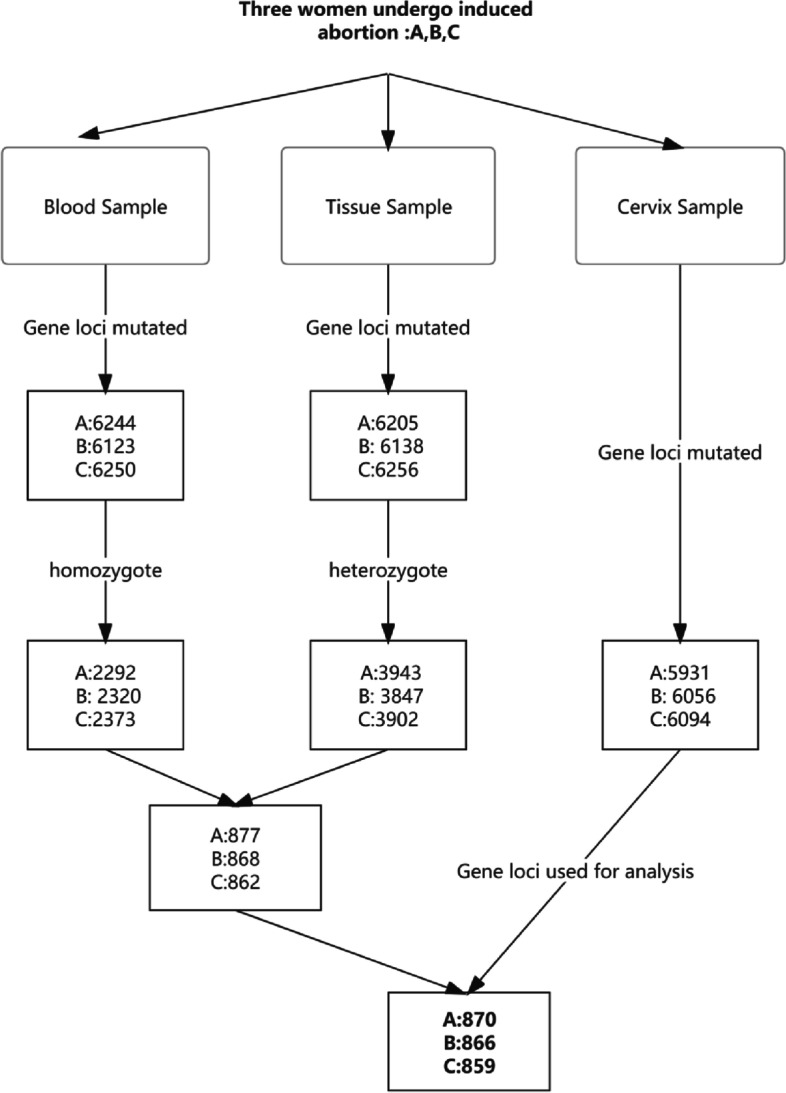
Flowchart of gene loci selection

As shown in Fig. 1, we conducted the selection for each patient individually. For patien A, we selected all the mutated homozygote gene loci in her blood sample (*n* = 2292). Then they were intersected with all the mutated heterozygote gene loci in abortion tissue A(*n* = 3943). Then we get a group of 877 gene loci that are heterozygote in the fetus but homozygote in the mother. As for the cervix samples, we filtered all the mutated gene loci(*n* = 5931). Finally we got an intersection of the 877 gene loci and 5931 gene loci for analysis(n-870). The final intersection represent the heterozygote mutaion happens only in fetus, not the mother. This rule out the influence of the mother’s signal in the cervix sample. The same selection was conducted for patient B and C. Patient A,B and C got 870, 866, 859 gene loci for analysis, respectively.

### Calculation of allele frequencies

The allele frequency in the blood sample was calculated by.

 

$$\text{Allele frequency in the blood sample}=\frac{Allele\ depth\ of\ the\ reference\ genotype\ in\ the\ blood\ sample}{Allele\ depth\ of\ the\ reference\ genotype\ in\ the\ blood\ sample + Allele\ depth\ of\ the\ variant\ genotype\ in\ the\ blood\ sample}$$

The allele frequency in the cervix sample was calculated by.

$$\text{Allele frequency in the cervix sample} = \frac{Allele\ depth\ of\ the\ reference\ genotype\ in\ the\ cervix\ sample}{Allele\ depth\ of\ the\ reference\ genotype\ in\ the\ cervix\ sample + Allele\ depth\ of\ the\ variant\ genotype\ in\ the\ cervix\ sample}$$

### SRY detection

There was a total of four Y chromosomal genomic intervals in our panel, which was confirmed to be exclusive for male fetuses. Then, we detected these four SRYs in the samples of patient A, the only patient who carried a male fetus.

## Results

**Fig. 2:**
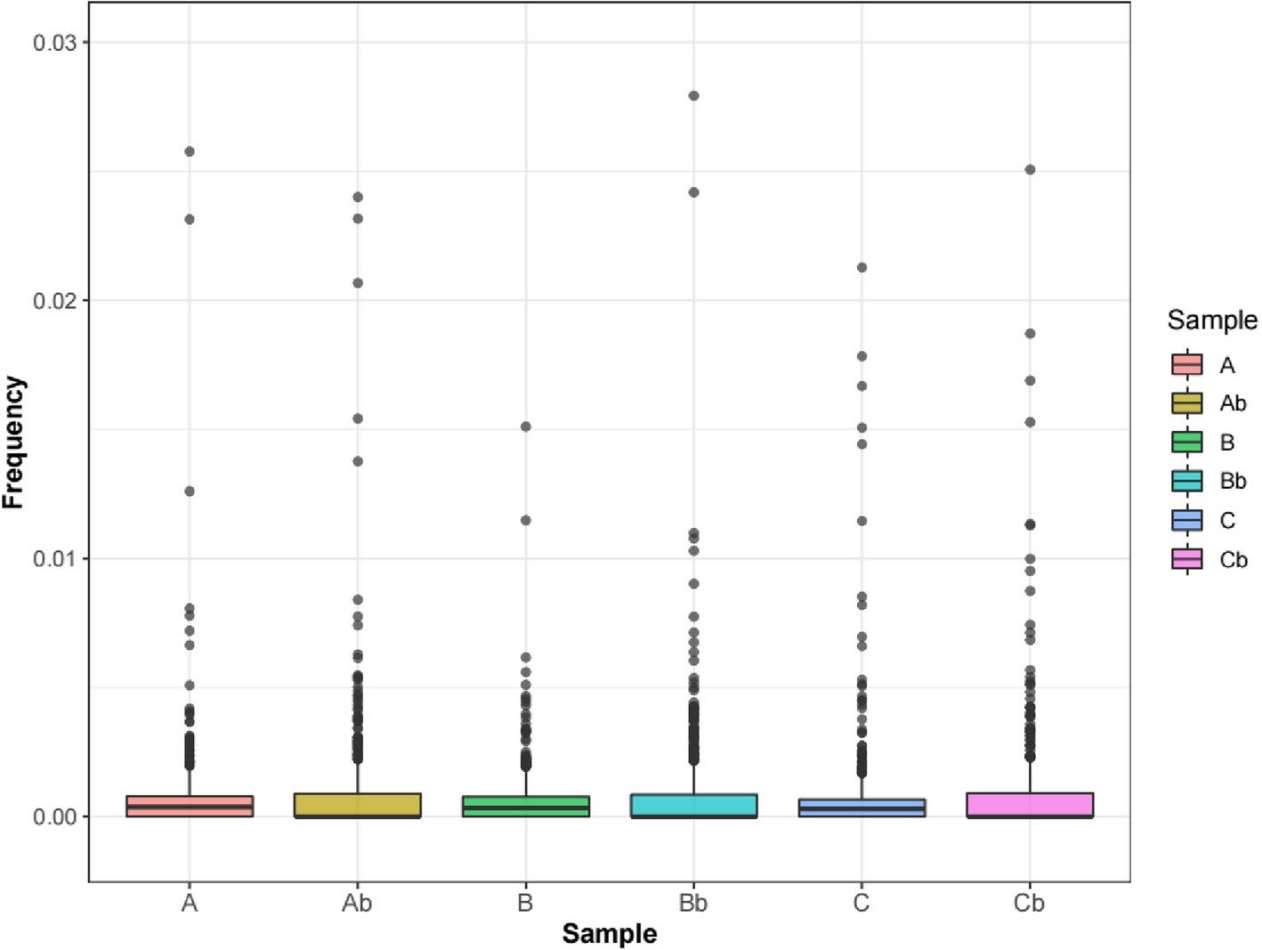
Allele frequencies in cervix and blood samples. The allele frequencies for the cerix and blood samples of patient A, B, C are shown. Abbreviations: A Cervix sample A, Ab Blood sample A, B Cervix sample B, Bb Blood sample B, C Cervix sample C, Cb, Blood sample C

**Fig. 3:**
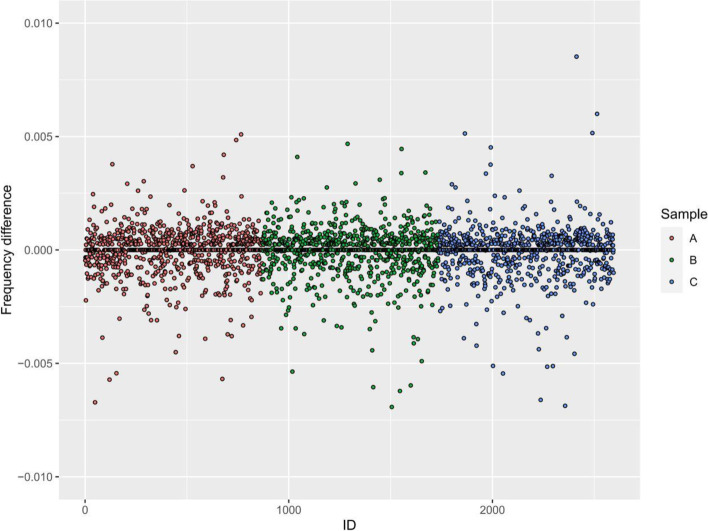
Differences of the allele frequencies between the cervix and blood samples. The differences of allele frequencies of each gene loci in the cervix and blood sample were plotted for each individual patient

In Fig. 2, we presented the allele frequencies of each gene loci in the cervix samples and maternal blood samples for three women. The differences of the allele frequencies between the cervix and blood samples were plotted in Fig. 3. The mean and median values were presented in Table 1. One gene loci corresponds to two alleles, so the estimated percentage of trophoblastic content from cervix samples was 0.243%, 0.192%, 0.186%, respectively. Based on the Mann-Withney-Wilcoxon test, p values for three women were all larger than 0.01 which does not indicate a significant difference.

**Table 1:** Allele frequencies and trophpoblast content in cervix

Sample	N	Avg.C %	Avg.B %	Med.C%	Med.B%	*P* value	%Trophoblast
A	870	0.122%	0.095%	0.037%	0	0.50	0.243%
B	866	0.096%	0.095%	0.036%	0	0.63	0.192%
C	859	0.093%	0.088%	0.030%	0	0.56	0.186%

*Abbreviations: N Number of gene loci for analysis, Avg.C%, Percentage of average allele frequency in cervix sample, Avg.B%, Percentage of average allele frequency in blood sample, Med.C%, Percentage of median allele prequency in cervix sample, Med.B%, Percentage of median allele frequency in blood sample, P value  P value of Mann-Withney-Wilcoxon for allele frequency in cervix and blood sample, % Trophoblast, estimated percentage of trophoblast from cervix samples*

We also detected the Sex Determining Region Y (SRY) in the samples of patient A, who carried a male fetus. As shown in Table 2, in the abortion tissue, the SRY signal was detected in all four regions. For the cervix sample, 4 reads were found to be matched for region chrY_p11.31_001_SRY_Y. No reads were matched for the other three SRYs.

**Table 2:** Signal for Sex Determining Region Y (SRY) for subject A

Sex Determining Region Y (SRY)	Raw data		
	Cervix	Blood	Abortion Tissue
chrY_p11.31_001_SRY_Y	4	0	3119
chrY_p11.2_001_rs4027562	0	0	375
chrY_q11.221_001_rs9786043	0	0	354
chrY_q11.222_001_rs3900	0	0	896

## Discussion

Previous studies had explored the use of trophoblast cells in prenatal testing, and the procedure typically involves two parts. To seperate trophoblast cells, immunomagnetic separation and flow cytometry is often used, but a certain amount of target cells may be lost due to highly specificified antibodies and antigen destruction caused by specimen pretreatment [8]. Laser Capture Microdissection (LCM) can also be used but the the cutting precision may lead to the damage of the nucleus.Differential adhesion method uses the different adherent speed of trophoblast cells and maternal contaminated cells to remove maternal suspended cells [8]. However, in Yuan et al.’s research, only 38% of fetuses’ gender was correctly identified. For trophoblast cells identification, immunohistochemical staining is usually used, but its efficiency is highly dependent on the specificity of antibody and antigen destruction [11]. Instead of using seperation before identification, we extract DNA directly and then perform the sequencing procedure. To a great extent, this avoids the possible loss of target cells.

For the “allele frequnecy” method, we filtered the gene loci that was mutated in the cervix cells, and homozygous in blood but heterozygous in the abortion tissue. This step rules out the the major maternal contribution to the fetus for our further analysis. However, the homozygous genotype does not necessarily represent a 100% homozygous signal. For example, geno loci X of a mother is homozygous with a 99.9% homozygous allele frequency with 0.1% heterozygous allele frequncy. Then, 99.9% homozygous signal and 0.1% heterozygous signal will be detected in all maternal cells, including blood and the cervix. Eventhough we filrtered the homozygous gene loci in the maternal blood and the heterozygous gene loci in cervix sample, the mother’s heterozygous signal in the blood and the cervix sample cannot be completely ruled out. To address this concern, we compared the allele frequncies of the cervix sample with those in the maternal blood. If the allele frequncies in the cervix sample are not significantly different from that in the blood sample, the signal detected in the cervix might come from the mother but not the fetus. If the signal we detected in the cervix cells is significantly different from that in the maternal blood, the contribution from the mother can be ruled out and the signal from the trophoblastic content is detected. The mean allele frequencies for the three subjects were 0.122%, 0.096% and 0.093% respectively. Since one gene loci has two alleles, the estimated trophoblast content was 0.243%, 0.192%, 0.186%, respectively. Although the signal was small, it was comparable to the previous study with a 1/2000 content of trophoblast [12]. However, as can be seen from Figs. 1 and 2, the allele frequencies in the cervix cells were very similar to those in the blood cells. We compared the allele frequencies in the cervix and blood cells using paired-test wilcoxon test. The P values were all larger than 0.1, indicating an insignificant difference of allele differences between the gene loci in the cervix and blood cells. Therefore, using the “allele frequency” method, the signal of trophoblastic cells was not detected successfully.

However, we successfully detected the SRY signal in a cervix sample, indicating a preliminary successful retrieval of trophoblast from the cervix canal. The finding was consistent with a recent study in Belgium [13]. However, we only had one male fetus, the sample size was too small and we aim to include more patients for further study before its clinical application.

## Conclusion

We successfully detected the SRY signal in a cervix sample, indicating a successful start of trophoblast retrieval from the cervix canal. Further study needs to be conducted to verify our finding and hopefully, the successful retrieval of trophoblast from the cervix canal will be applied to the early detection of fetal chromosomal abnormalities by NIPT.

## Data Availability

The datasets used and/or analysed during the current study available from the corresponding author on reasonable request.
